# Microfluidics for Peptidomics, Proteomics, and Cell Analysis

**DOI:** 10.3390/nano11051118

**Published:** 2021-04-26

**Authors:** Rui Vitorino, Sofia Guedes, João Pinto da Costa, Václav Kašička

**Affiliations:** 1 UnIC, Departamento de Cirurgia e Fisiologia, Faculdade de Medicina da Universidade do Porto, 4785-999 Porto, Portugal; 2 iBiMED, Department of Medical Sciences, University of Aveiro, 00351234 Aveiro, Portugal; 3 LAQV/REQUIMTE, Department of Chemistry, University of Aveiro, 00351234 Aveiro, Portugal; sguedes@ua.pt; 4 Department of Chemistry & Center for Environmental and Marine Studies (CESAM), University of Aveiro, 00351234 Aveiro, Portugal; joao.pinto.da.costa@gmail.com; 5 Institute of Organic Chemistry and Biochemistry of the Czech Academy of Sciences, Flemigovo n. 542/2, 166 10 Prague 6, Czech Republic

**Keywords:** cell sorting, LOC, microchip electrophoresis, microfluidics, microTAS, peptides, peptidomics, proteins, proteomics, single-cell analysis

## Abstract

Microfluidics is the advanced microtechnology of fluid manipulation in channels with at least one dimension in the range of 1–100 microns. Microfluidic technology offers a growing number of tools for manipulating small volumes of fluid to control chemical, biological, and physical processes relevant to separation, analysis, and detection. Currently, microfluidic devices play an important role in many biological, chemical, physical, biotechnological and engineering applications. There are numerous ways to fabricate the necessary microchannels and integrate them into microfluidic platforms. In peptidomics and proteomics, microfluidics is often used in combination with mass spectrometric (MS) analysis. This review provides an overview of using microfluidic systems for peptidomics, proteomics and cell analysis. The application of microfluidics in combination with MS detection and other novel techniques to answer clinical questions is also discussed in the context of disease diagnosis and therapy. Recent developments and applications of capillary and microchip (electro)separation methods in proteomic and peptidomic analysis are summarized. The state of the art of microchip platforms for cell sorting and single-cell analysis is also discussed. Advances in detection methods are reported, and new applications in proteomics and peptidomics, quality control of peptide and protein pharmaceuticals, analysis of proteins and peptides in biomatrices and determination of their physicochemical parameters are highlighted.

## 1. Introduction

**Figure 1:**
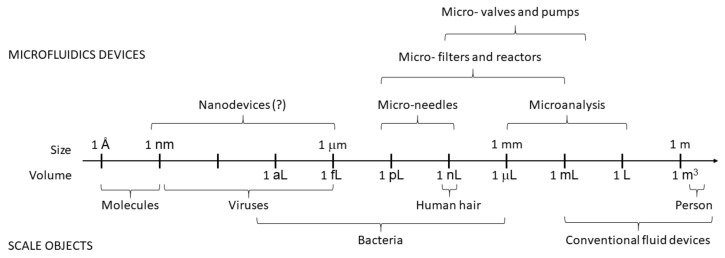
Scales and volumes in microfluidics.

Microfluidics (MF) is a relatively new branch of science and microengineering that deals with manipulating fluids in microchannels with at least one dimension of 1 to 100 micrometers [1,2], as shown in Figure 1. Scientists consider it a new discipline not only because of the recent emergence of microfluidic (MF) devices that can implement rapid solutions to complex analytical problems at the microscale but also because the physical principles of fluid flow at such small length scales differ from those in macrosystems [3]. MF spans several disciplines—physical and chemical sciences, micromechanics, electronics, and mechanical engineering. It has wide applicability in many fields, with particular emphasis on biology, biochemistry, biotechnology, medicine, pharmacology, and food and environmental analysis. It has made great progress in the last 15 to 20 years [4]. Intensively developing research areas in MF are lab-on-chip (LOC) devices [5,6] and microanalytical systems (μTAS) [7]. They can be considered synonymous with integrated circuits in electronics. The MF technology is based on micropumps, mixers, filters and valves to realize chemical and biological laboratory processes on a single chip. MF requires tiny samples and reagents for analysis, which makes it environmentally friendly and minimally invasive [8].

MF is perceived as a new platform for highly efficient separation and highly sensitive analysis of (bio)molecules and (bio)particles in biochemistry, molecular biology and biotechnology [8] and relies on scale reduction to reduce material consumption and cost [1]. MF exploits the potential of flowing liquids at the microscale to generate “quantitative assays”. In peptidomics and proteomics, MF is most commonly used in combination with MS analyses. Hydrophobic membranes were originally used to adsorb native peptides or peptides generated by enzymatic digestion of proteins, followed by their desalting and elution in a controlled environment for MS analysis [1,2]. Peptides and proteins are analyzed at MS mainly by two ionization techniques, electrospray ionization (ESI) and matrix-assisted laser desorption ionization (MALDI). ESI–MS and MALDI–MS are used not only to determine the relative molecular masses of peptides and proteins but also to elucidate their amino acid sequences and post-translational modifications [9,10,11,12]). Rapid and highly sensitive analysis was achieved by using ultralow sample volumes and amounts (picomoles to femtomoles of compounds in nanoliters to picoliters of volume per single analysis), high separation efficiency, and short analysis times. The widespread use of MF can be attributed to the inherent advantages of MF instruments: Wide applicability, compactness and the need for extremely low sample volumes of the analyzed compounds or particles, as well as low reagent consumption [2,12,13].

This review article, after a brief introduction to the fundamentals of MF, focuses mainly on the recent developments and applications of MF in the fields of peptidomics, proteomics, and cell separation and analysis. In combination with MS and other novel detection methods, MF targets clinical problems related to disease diagnosis and therapy. In addition, we describe several technological advances in the different areas of MF, which optimize the separation and analysis of peptides, proteins and cells [14,15].

## 2. Fundamentals of Microfluidics

### 2.1. Microfluidic Chips

**Figure 2:**
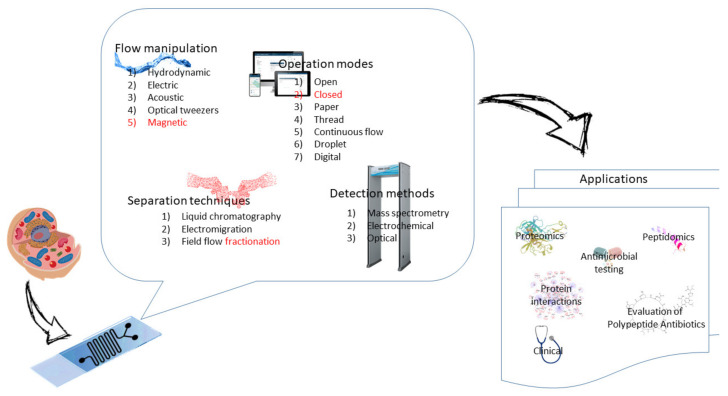
Flow manipulation, operation, separation, detection methods and applications of microfluidics.

MF chips are devices for processing or viewing minute amounts of liquid in the microliter to picoliter range [16]. MF chips (Figure 2) consist of very thin internal microchannels (with 1–100 μm thickness). They have connections to the outside through holes on the chip called inlet and outlet ports. These chips are made of thermoplastics, such as glass, fused silica, acrylic (poly(methyl methacrylate)) (PMMA), or poly(dimethylsiloxane) (PDMS), a form of transparent silicone rubber” [16,17]. The chips are usually transparent and have an overall length or width of 1 to 10 cm. The overall thickness of the chips varies from about 0.5 mm to 5 mm.

#### 2.1.1. Glass vs. Plastic Chips

At the beginning of MF research, these devices were mainly made of glass or silicon. The choice of materials was limited as the chips were mostly made by photolithography. The main advantages of these materials were accurate dimensional control during fabrication, good resistance to water and organic solvents, and suitable surface properties for electrophoretic and chromatographic applications. In particular, glass and fused silica have been highly valued due to their amorphous nature materials and possessing both electrically insulating and optically transparent properties [16]. On the other hand, the silicon substrate is relatively expensive and optically nontransparent, limiting its application for optical detection in (bio)chemical and biomedical analysis. MF chips are now prototyped and fabricated using laser micromachining techniques, allowing a wider range of material options. Organic polymers have emerged as viable materials compared to glass or silicon, as they have better material properties and are cheaper to process as well as to purchase [16,17].

#### 2.1.2. Fabrication Process of the Chips

Most of the fabrication methods of MF involve technologies adapted from the production of semiconductor integrated circuits (IC) and microelectromechanical systems (MEMS). These methods are mainly based on photolithography [18]. This technology provides high-resolution results but requires a clean environment and significant upfront investment and maintenance. Alternatives to photolithography include laser cutting, paper wax, vinyl cutter, Norland optical adhesive, and shrinkable polymer, to name a few [19,20,21,22,23,24,25]. However, these methods cannot achieve high-resolution results. 3D printing also could not overcome this problem due to its low throughput and resolution. Although it is a scalable, robust and cost-effective method, its main drawback is related to the biocompatibility of the resin. Other methods require using SU-8 components, which can be quite complicated and expensive. The high cost associated with SU-8 can be avoided by using methacrylate (MA) with low shrinkage in MF environments. The master mold is used as the mold for fabricating the polymer device. Chips are fabricated using PDMS, but the choice of prepolymer brings significant drawbacks in terms of difficulty scaling for mass production, protein adsorption, reverse hydrophilicity, and contamination of “cyclic silicone monomer derivatives” [26,27,28,29]. Plastic chip MF are usually fabricated by making small depressions or fine grooves on the material’s surface (layer), followed by enclosing these depressions using a second layer that forms chambers or microchannels [16]. These chambers or channels must be well sealed to ensure that they are tight. Depending on the choice of material, the channels are created by etching, hot stamping, injection molding, micromachining, or soft lithography. MF chips are often fabricated by 3D printing, but this process has serious shortcomings in terms of material selection, minimum feature size, optical transparency, or surface roughness [16,17].

Photolithography provides high-resolution results but requires a clean environment and significant upfront investment and maintenance. Alternatives to photolithography include laser cutting, paper wax, vinyl cutter, Norland optical adhesive, and shrinkable polymer, to name a few [19,20,21,22,23,24,25]. However, these methods cannot achieve high-resolution results. 3D printing has not been successful in this case due to its low throughput and resolution. Although it is a scalable, robust and cost-effective method, its main drawback is related to the biocompatibility of the resin. Other methods require using SU -8 components, which can be quite complicated and expensive. The high cost associated with SU -8 can be avoided by using methacrylate (MA) with low shrinkage in MF environments. The master mold is used as the mold for fabricating the polymer device. Chips are fabricated using PDMS, but the choice of prepolymer brings significant drawbacks in terms of difficulty scaling for mass production, protein adsorption, reverse hydrophilicity, and contamination of “cyclic silicone monomer derivatives” [26,27,28,29].

The application of LED-UV light and MA gels for microchip fabrication resulted in higher reproducibility and accuracy of the fabricated MF devices. The method requires minimal training and cost [27,30]. The fabrication process involves creating the photomask, followed by the photolithography process, consisting of coating with MA, exposure to UV light, and washing. MF chips are formed by soft lithography and soft embossing. These methods are accessible and affordable and can be used to manufacture MF devices even in low-resource environments. By eliminating specialized equipment, such as plasma machines, spin-coaters, and mask-exposers, costs could be reduced tenfold. The researchers concluded that rapid development of MF is possible if a wide range of tools can be prototyped without incurring significant costs to set up and operate the equipment [27].

To address the problems associated with high-cost MF devices, low-cost MF fabrication has been proposed [31]. Nguyen and colleagues (2018) [27] recommended an MF fabrication method that provides high sensitivity and lowers cost by reducing the number of reagents and analysis time. The technology is called Lab-on-a-Chip (LOC) and can be used to detect infectious agents in low-resource settings. The conventional process of MF prototyping consists of two steps: creating the “master mold” and its replication into an MF prototype made of polymers. In this process, high costs are associated with the photolithography method used to create the master molds. For mass producing MF chips, mature manufacturing processes, such as injection molding and plastic welding, are available to produce low-cost disposable chips. Even lower costs can be achieved with paper-based MF devices.

### 2.2. Flow Manipulation

**Figure 3:**
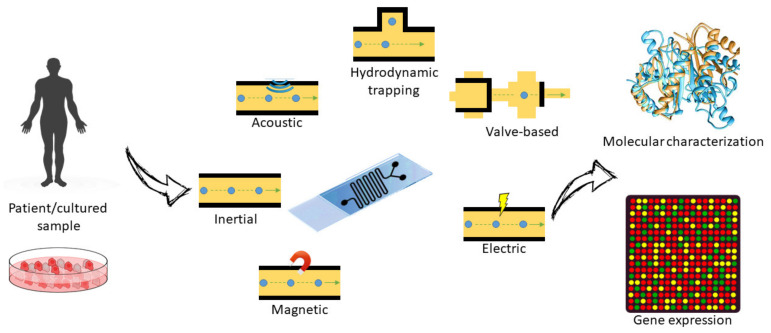
Flow manipulation.

The flow of fluid in microchannels and the movement of microparticles (MPs) and nanoparticles (NPs) present in the fluid are usually generated hydrodynamically or electrically. In addition, the fluid flow and the movement of MPs and NPs in microchannels can also be generated by using acoustic, optical, and magnetic forces, see Figure 3.

MF technology can precisely process small volumes of fluid [32]. This is important for both processing and delivery of (bio)molecules and (bio)particles for detection. MF can also increase the mass transfer rate and thus increase the rate of fast chemical processes associated with detection. This feature is particularly important for sensors that require binding reactions on solid surfaces (e.g., protein and DNA chips) [33].

In various applications, such as bioanalysis, diagnostics, drug delivery, and self-cleaning surfaces, the manipulation of MPs and NPs is necessary [34]. The rapid development of micro- and nano-engineering has promoted the rapid development of various technologies for manipulating MPs and NPs, including both established techniques and currently developed innovative early-stage techniques. Some of the recently developed techniques include artificial cilia and microbots [33].

#### 2.2.1. Hydrodynamic Methods

The hydrodynamic flows of fluids and the movement of MPs and NPs contained in these fluids in a complex network of microchannels of the MF device are controlled by the pressure supplied by an external tank or by the peristaltic or piston pumps attached to the device [32] and by balancing the opposing lateral forces acting on the fluid and particles moving along the MF channels [35,36]. Alternatively, the hydrodynamic flow is relatively frequently generated by the centrifugal forces using microchannels on the disk, the revolutions of which are controlled by a computer CD driver. On the other hand, capillary and gravitational forces are only rarely employed in MF devices.

#### 2.2.2. Electric Methods

Fluid flow is often generated by an electric field applied from a high voltage power supply placed outside but close to the MF device. The electric field can manipulate the flow of the fluid and the NPs and MPs inside the microchannels of the MF devices by electroosmosis, electrophoresis, or dielectrophoresis [37]. The advantage of electroosmotic flow is that it is a laminar flow with a piston-like profile of velocities, resulting in less dispersion of separation processes. Electric methods belong to the most important driving forces in MF, especially in fluids with charged solutes and particles. They are frequently used for pumping, mixing, separation, sorting and analysis of peptides, proteins and cells by liquid chromatographic and electromigration methods. The disadvantage of electric methods is the electrolysis in the electrode compartments of MF devices.

#### 2.2.3. Acoustic Method

The acoustic method for manipulating fluid fluids and (bio)particles (referred to as acoustic tweezers) provides a noncontact way of processing fluids and particles [38,39]. The basic theory of particle manipulation with acoustic tweezers shows that particles tend to collect at the pressure nodes or anti-nodes of acoustic waves under the influence of the acoustic radiation force acting on the particles [39]. Acoustic methods are applied for sample introduction in automated systems for activity testing of a large set of compounds.

#### 2.2.4. Optical Methods

Optical forces (radiation pressure or optical tweezers) are based on converting light energy to liquid motion. They are featured by their contactless spatial (micrometric spots) and temporal (ultrafast) control, and dynamical reconfiguration. They proved to be useful in the manipulation of suspension in a single fluid phase. Optical tweezers were developed by Chu et al., (1986) [40]. They involve using tightly focused laser beams to capture and manipulate particles or cells with very high precision [41]. These techniques are promising for future MF developments, but their application for real-world samples in peptidomics, proteomics and cell analysis is still limited.

#### 2.2.5. Magnetic Methods

Magnetic methods use magnetic fields generated by permanent magnets or electromagnets to manipulate fluid liquids and particles [42,43]. In a nonuniform magnetic field, magnetic or magnetically labeled particles/cells are attracted to the larger magnetic field. The magnetic force depends on the magnetic field gradient but also on the size and magnetic properties of the fluids and particles/cells. Like electric fields, magnetic fields can be applied for pumping, mixing, and sorting charged compounds or magnetic particles in chemical and biological analyses.

### 2.3. Basic Modes of Microfluidics

MF can be divided into several types:

Open MF is also referred to as open surface or open space MF, in which the solid support for the fluid flow is removed from at least one spatial boundary. This means that the resulting fluid flow is exposed to air or other boundary surfaces, such as another fluid [44].

In closed MF, the flow is confined by fixed supports on all longitudinal sides of the microchannel(s). The flow is usually driven by pressure generated by micropumps (e.g., syringe micropumps) or by an electric field. By eliminating the need for external pumping, the cost of the process can be reduced, allowing any laboratory to use this device with pipettes [45].

Paper MF takes advantage of the paper’s unique property of conducting liquid from an inlet to the desired outlet through capillary effects. This is a promising method because the paper has a low environmental impact, is widely available, and is inexpensive. It is also versatile as it is available in different pore sizes and thicknesses. Various coatings, such as wax, are commonly used to guide the fluid flow in this type of MF system [46].

Thread MF is similar to paper MF and takes advantage of the wicking properties of the thread. Commonly used materials include hemp, nitrocellulose, nylon, polyester, viscose, silk and wool. The versatility of this type of MF is well-known, as specific patterns can be formed by interweaving the threads [47].

Continuous flow MF manipulates the flow of fluid through the fabricated microchannels without affecting the continuity of the flow. External sources (micropumps) are used to fabricate the fluid flow. Some of the commonly used pumps are syringe or peristaltic pumps. Fluid flow can also be maintained by internal mechanisms, such as capillaries, magnetic or electrical forces [48].

Droplet MF (DrMF) has recently emerged as a potent new mode in MF technology [34]. MF Droplet devices produce droplets with small volumes (in the range of nanoliters to femtoliters). This technique is used to manipulate discrete volumes of liquid in immiscible phases. Laminar flow and Reynolds number are kept low. This method is currently receiving much attention. DrMF uses two immiscible phases called the dispersed phase and continuous phase (the former is the droplet phase, and the latter is the droplet flow medium) [49]. Three main applications of DrMF are microbial research, microparticle synthesis and molecular biology [34].

In microbial research, microorganisms are encapsulated in the droplets to analyze their response to various drug discovery reagents [33]. In the study of microparticle synthesis, the droplets are usually made of hydrogels, and various techniques are used to solidify them after preparation, such as thermal, chemical, or light photographic techniques. In molecular biology, the droplets serve as bioreactors. Individual cells are captured in the droplets and undergo a series of reactions that allow each droplet to be analyzed individually [9].

Digital MF (DMF) is based on manipulating microdroplets by external electrical and magnetic forces [49,50,51]. In this system, droplets are analyzed, dispensed, mixed, moved, reacted or stored on a platform with multiple sets of isolated electrodes. It can be considered an alternative method of droplet analysis MF and provides a step-by-step process for analyzing specific volumes of liquid [52].

Electric potentials are applied to an electrode array coated with a hydrophobic insulator. An electric potential causes a charge to accumulate on each side of the insulator. This approach allows precise manipulation of droplets (moving and mixing, splitting and merging, and dispensing from reservoirs). DMF helps overcome the pitfalls associated with microchannel-based fluidics by providing control over solid and liquid reagent phases. This novel method allows one to work with heterogeneous systems by eliminating the possibility of clogging or the need for cumbersome microvalve networks. DMF approaches can be either open (using a single substrate) or closed (using two substrates with droplets in between). DMF suffers from nonspecific adsorption on the surface, which can lead to sample loss, sticking of droplets or cross-contamination. The problem can be solved by using immiscible oils, low concentration amphiphilic polymer additives or removable hydrophilic insulators, depending on the situation. DMF has a number of applications related to the analysis of cell-based assays and DNA [50,51]. It is also a valid method for various other clinical applications [51].

### 2.4. Separation Techniques Implemented in Microchips

#### 2.4.1. Liquid Chromatographic Methods

Since liquid chromatography (LC) is one of the most effective analytical separation techniques, the requirements for its miniaturization are particularly high. The pressure and/or electroosmotic flow (EOF) driven LC and electrochromatography (EC) in the MF chips with the continuous beds was introduced in 2000 by Hjerten et al. [53,54]. Since then, various structures and implementations of LC separations have been developed in the MF format. The design, developments and applications of LC chips have attracted increasing attention. Compared with conventional LC columns, LC MF chips offer several advantages: smaller sample and reagent volumes, fast and inexpensive processing, compounds without dead volume, and the possibility of multiplex analysis. Therefore, miniaturization of LC systems is a clear goal for the fabrication of the LC MF chips with a focus on particle-based LC chips. In the last 15 years, the fabrication of these LC chips has been a popular scientific trend in MF technology [54]. The hurdle is to develop a reproducible and efficient process to fabricate micro-packages. Most of the works have dealt with particle-filled LC capillaries and chips [55,56]. However, the typical techniques used to prepare chromatographic columns could not be readily implemented at the microscopic level. Moreover, the design of (sub)nanoliter sample injection and sensitive detection is still a challenge in developing the new advanced LC-based MF systems [55].

The best separation efficiency was achieved with commercially available LC MF chips, especially for use in unique instruments developed by related companies. To improve the robustness of LC MF chips, advanced research in various fields and appropriate subsequent integration in side operations are widely needed. Obviously, in the next decade, the attempt to effectively miniaturize analytical instruments will continue.

LC MF chips need to be coupled with MS detection to obtain relevant properties of the separated analytes, especially peptides and proteins. Depluverez et al. [57] used the LC chip in conjunction with multiple reaction monitoring (MRM) MS detection to measure protein virulence factors of *Burkholderia* cenocepacia, an opportunistic pathogen commonly isolated from cystic fibrosis (CF) patients. Several virulence factors have been described, including extracellular enzymes secreted by secretion systems of the II and VI types. It is expected that MS detection will be frequently combined with MF LC separations in the future. A large number of small sample batches can be analyzed with high throughput. MF LC chips can be considered low-cost or even disposable systems.

A highly integrated LC microchip with a LED-based absorbance detector was used for the rapid and accurate determination of glycated hemoglobin (HbA(1c)) [58]. The good linearity (R^2^ = 0.9860) and low limit of detection (LOD) (about nine times lower than the commercial detector), inaccuracy of less than 4.3% and repeatability (=2.8%), which are comparable to the results of a commercial LC device, enabled the measurement of HbA1c levels of patients with diabetes. The developed MF LC system showed great potential for accurate point-of-care diagnostics in diabetes.

A new two-dimensional chip-based high-performance liquid chromatography (2D-chip HPLC) system was developed by the Belder group [59]. By combining injection, separation and detection on a dead-space-free fused silica chip, all peak dispersion effects outside the column could be minimized. The use of intrinsic fluorescence with excitation in the deep UV range and electrospray ionization–mass spectrometry (ESI–MS) as detection modes after the first and second separation dimensions, respectively, enabled label-free analysis of complex samples, including tryptic digests of proteins

TiO_2_ nanoparticle-packed microchannel array glass microchips fabricated by a plasma-assisted method for precise microstructure alignment were used for selective enrichment of phosphopeptides from a protein digest mixture, demonstrating the high capacity and selectivity of in-tube solid-phase microextraction (SPME) microchips [60].

Recent advances and future prospects in component fabrication, detection methods, and commercial implementation for the analysis and microscale isolation of proteins in MF devices by electrophoretic and chromatographic methods based on protein differences in size, charge, affinity, and mobility have been discussed by Rodriguez-Ruiz et al. [61].

MF chips with reverse-phase monoliths (RP) that enable both solid-phase extraction (SPE) and on-chip labeling were presented by Nge et al. [62]. The method integrated on-chip enrichment of samples with fluorescent labeling and purification. Polymer monoliths were prepared from butyl methacrylate using cyclic olefin copolymer (COC). The retained sections were then labeled with fluorogenic probes Alexa Fluor 488 and Chromeo P503. SPE was used in advanced sample preparation, including extraction, purification and enrichment. Monolithic columns are commonly used to be easily mounted on a chip without the need to maintain structures, such as frits. RP-based columns are useful for the extraction of nonpolar to slightly polar compounds. COC is the suitable polymer substrate for SPE microchips due to their resistance in organic solvents.

Off-chip sample preparation was a major challenge for the miniaturized study. The proposed method integrated sample enrichment with on-chip labeling and purification. Combining this approach with affinity extraction provides an additional dimension of specificity and enables an efficient and automated bioanalysis process.

Recent achievements in sample preparation for protein determination using solid supports (microbeads, monolithic materials and membranes) in MF devices have been critically reviewed by Dziomba et al. Selective extraction and preconcentration of target proteins and peptides, especially from biological fluids, is of paramount importance for the successful detection and quantification of these biopolymers [63].

#### 2.4.2. Electromigration Methods

Capillary electrophoresis (CE) and microchip electrophoresis (MCE) has evolved over the past 30 years into high-performance separation techniques that are widely used for the analysis and physicochemical and biochemical characterization of peptides, proteins, and other (bio)molecules and (bio)particles. They have high separation efficiency, high sensitivity to mass (amount of substance), short analysis time, and require extremely small sample volumes and quantities, as well as small amounts of reagents and solvents. The methods of CE include (i) pure electrophoretic techniques, such as zone electrophoresis (CZE) in free solution or sieve (gel) medium (CSE or CGE), isotachophoresis (CITP), isoelectric focusing (CIEF) and affinity electrophoresis (ACE); and (ii) combined electrochromatographic techniques, electrokinetic chromatography (CEKC) and electrochromatography (CEC). They are now considered as recognized counterparts or complements to the most widely used separation methods, the various modes of HPLC and UHPLC.

Of the above CE/MCE methods, CEC, which separates analytes according to their electrophoretic mobilities determined by the charge/size ratio in a continuous background electrolyte (BGE), is the most widely used. CITP with the discontinuous electrolyte system, consisting of the so-called conducting and final electrolytes, is mostly used for the concentration of dilute analytes. CIEF separates amphoteric analytes, especially peptides and proteins, according to their isoelectric point (pI). ACE is used for the selective analysis of specific compounds in complex mixtures and for the study of (bio)molecular interactions. CEKC and CEC are used to analyze charged and uncharged compounds. CE and MCE methods are usually coupled with UV absorption, LIF, and MS detectors to identify and quantify the separated analytes.

However, CE and MCE methods still suffer from some shortcomings. Adsorption of analytes on the inner wall of fused silica capillaries or glass or polymer microchannels is a major problem. This problem is solved by different types of coatings. Depending on the bonding type of the coating material, capillary wall coating can be divided into the dynamic, permanent or semipermanent form [64]. In dynamic coating, electrolyte-soluble agents can be introduced into the BGE to protect the capillary walls during separation. The permanent coating is irreversibly bound to the inner surface by physical adsorption or covalent bonding [65]. In semipermanent coating, the adsorbed coating material must be renewed after several CE passes.

Various materials and technologies have been developed for the coating of capillary and microchannels [66,67]. The use of nanomaterials as capillary coating agents opens new opportunities to improve the separation efficiency and selectivity of the separation process [68,69]. Variable coatings can also be used to change the magnitude and direction of electroosmotic flow (EOF) [70,71].

Another disadvantage of CE and MCE is the relatively low concentration sensitivity due to the ultrasmall injected sample volume (nano-picoliter range) and the short optical path of the most commonly used UV-vis spectrophotometric detector. This drawback is compensated by various enrichment methods based on chromatographic or electromigration principles; for their description, see the recent reviews [72,73].

The combination of SPE with capillary electrophoresis (CE) or microchip electrophoresis (MCE) offers increased sensitivity and purification of the sample. Labeling is usually done off-chip, but on-chip labeling also has been performed in both pre- and post-column configurations. Fluorogenic reagents were used for on-chip formulations to increase the higher sensitivity of laser-induced fluorescence (LIF).

Sensitivity improvements of more than three orders of magnitude were achieved using these concentration techniques. However, more effective concentration techniques are still needed.

Despite the great advances in CE /MCE methods, classical slab gel electrophoresis is still widely used for protein analysis in biochemical and clinical laboratories. It is desirable to expand using CE /MCE in these laboratories to increase the speed and throughput of protein/peptide analysis, especially for large proteomic/peptidomic studies. The commonly used Western blotting technology can also be performed in CE and MCE format [74,75].

In the future, intensive research and development of MCE methods and their extended application in the analysis of peptides, proteins and other (bio)molecules is foreseeable [76].

#### 2.4.3. Field-Flow Fractionation

Since Giddings proposed the field flow fractionation (FFF) method in the 1960s [77], a number of FFF-based techniques have been reported for the separation of (bio)macromolecules and (bio)particles in chip-based laboratory systems MF [78]. Continuously loaded single-phase field forces require external forces or use only inertial shear forces.

Depending on the direction of the applied force, the separation force can be tangential or perpendicular to the flow direction and can take the form of batch or continuous loading techniques. In batch separation technique, particles follow the same path but at different velocities and fractionate only over time [55]. Therefore, these methods require a precise injection of very small amounts of sample into the separation channel. In another case, the applied force has a component perpendicular to the flow direction so that the particles move laterally and separate in space [79].

Several methods have been developed so far. These methods use external forces, but in each case, the particular characteristics and properties of the unit must be considered. The need for external forces increases the complexity of the equipment and may limit using certain reagents (e.g., biological samples) [80]. Comparing the performance of integrated sample classification methods is not always straightforward. Many methods offer high throughput, while others offer high resolution. Many MF devices are easy to use, while other technologies may require trained personnel [43]. Many separation principles require labeling of the components of the sample, while some methods are based on the inherent properties of the sample. As always, the best method depends on the sample and the analytical task to be performed [43].

Asymmetric flow FFF (AF4) is a widely used and universal technology in the FFF family, and its publications are rapidly increasing [81]. It is a sensitive separation and characterization technique that minimizes nonspecific interactions and enables wide separations from a few nanometers to micrometers, as well as excellent characterization of homogeneous and heterogeneous systems. In particular, coupling with multi-angle light scattering provides comprehensive access to sample properties. Information on molar mass, polydispersity, size, shape/integrity or density can be obtained almost independently of the materials used.

Shendruk et al. [82] used FFF to fractionate the entire range of spherical particle sizes. Their results show that FFF in normal mode in microfluidic channels is still a highly selective technology that can be achieved by simplified channel design.

### 2.5. Detection Schemes

Various methods are available to detect (bio)molecules and/or (bio)particles in the systems of MF. The physicochemical properties of the target analyte(s) determine the choice of the detection method.

Mass spectrometry (MS) is the most powerful detection mode, coupled online or offline with MF instruments. MS has several advantages: it is both universal and selective and is a highly sensitive detection mode capable of not only detecting but also identifying and quantifying analytes leaving MF microchannels. ESI and MALDI are the two most common methods for ionizing large (bio)molecules, especially polypeptides and proteins. Therefore, recent developments at the interface between MF microchips and mass spectrometers have focused on these two ionization methods [57,83,84].

Optical detection methods are among the most important and widely used detection methods in MF. In this broad category, fluorescence and especially laser-induced fluorescence (LIF) detection is a widely used detection method, along with less commonly used UV-vis spectrophotometric detection. Since MF devices can be coupled relatively easily to fluorescence detectors or fluorescence microscopes, the fluorescence of analytes is often used for their detection [84]. Recent developments in fluorescence detection in MF include fluorescence lifetime imaging, high-throughput single-molecule imaging, multicolor analysis, enhanced surface Raman spectroscopy, and surface plasmon resonance (SPR) detection [85,86].

Fluorescence is the most commonly used read mode in droplets MF due to its optical signal and fast time response. Applications such as improved multiplex analysis, enzyme development, and cell sorting require detecting two or more fluorescent colors. Standard multicolor detection systems that couple free-space laser light to an epifluorescence microscope are bulky, expensive, and difficult to maintain [87].

A typical detection station for droplets MF is based on an epifluorescence microscope and requires a complex light processing scheme to couple the excitation light from the laser into the free space of the microscope and focus it on the sample. After fluorescence is emitted from the droplet, the emitted fluorescence is filtered so that each detection channel uses a photomultiplier tube centered on the wavelength band [88]. An optical inspection system based on an epifluorescence microscope presents an entry barrier due to cost, complexity, and required maintenance. Since the optical fiber can be manually inserted into the device MF, optical fiber provides a method for building a simplified and powerful detection scheme, eliminating the need for a mirror-based optical path and allowing the optical path to be connected to a fiber optic connector [87].

Electrochemical detection has become a popular detection mode in MF due to its small size, high sensitivity, and selectivity. It is particularly used in portable MF devices on the microchip platform [89,90]. These simple, easy-to-use and sensitive analytical tools are essential for on-site detection of variable analytes in complex samples (such as food, feed, biological or environmental matrices) [91,92].

## 3. Applications of Microfluidics

### 3.1. Analysis of Peptides and Proteins; Peptidomics and Proteomics

#### 3.1.1. Introduction

Peptides and proteins are extremely important biological molecules. As hormones, hormone or drug receptors, enzymes, coenzymes, enzyme substrates or inhibitors, antigens, antibodies, immunomodulators, antibiotics, structural elements and transport molecules play a crucial role in all living organisms. They ensure the basic operations of the cellular machinery. In addition, there are many peptide and protein-based drugs and prodrugs, and some peptides and proteins are used as biomarkers [67]. Moreover, a complete analysis of all peptides (peptidomes) and proteins (proteomes) of a cell, tissue, biofluid, organ or organism is important to understand normal and pathological processes. This is the subject of peptidomics and proteomics—comprehensive and large-scale studies of complex mixtures of peptides and proteins. In this context, the relevance of peptides is increasing because the structure and function of proteins are often identified by their enzymatically generated peptide fragments. This bottom-up or shot-gun approach is one of the main directions in current proteomics research.

The separation and study of peptides and proteins in complex biological matrices is a challenging process that requires advanced and accurate methods that can provide relevant information about their structural and functional properties.

It has been known for more than a century that proteins can move or even “fly” under the influence of an electric field [93]. Therefore, electromigration methods represent powerful tools for their separation and analysis. Polyacrylamide gel electrophoresis in the presence of sodium dodecyl sulfate (SDS–PAGE), isoelectric gel focusing (IEF), and two-dimensional gel electrophoresis (2-DE), which combines the orthogonal principles of narrow tube gel IEF in the first dimension and plate gel SDS–PAGE in the second dimension, have been widely used in the past for protein and polypeptide analysis [94,95]. Currently, HPLC and UHPLC combined with high-resolution MS detection are the leading techniques for peptide and protein analysis [96]. Capillary and microchip electromigration methods (CE/MCE) are also very powerful and useful methods for the analysis and characterization of peptides and proteins [97,98]) and for applications in peptidomics and proteomics [99]. CE and MCE methods have several advantages, such as high separation efficiency, short analysis time, low sample and reagent consumption, and different separation modes (ZE, ITP, IEF, AE, EKC, and EC). In the field of peptidomics and proteomics, CE and MCE are usually combined with MS detection, as MS can identify and quantify the separated peptides and proteins. In addition to MS detection, UV absorption or LIF detection are also commonly used in CE and MCE analyses of peptides and proteins [98,100].

Biological samples (e.g., body fluids, tissues, and food extracts) contain complex mixtures of variable compounds with low and high molecular mass. Therefore, the target peptides and proteins usually need to be extracted or pre-isolated and/or preconcentrated from the sample matrix before analysis [101,102]. The method of sample preconcentration is based on (i) the principle of electrophoresis [103,104,105] (on-site field-assisted sample stacking, micelle stacking, ITP and IEF) or (ii) selective adsorption/extraction method (elution) [106,107]. Usually, a combination of different types of preconcentrates is used. Another important issue in CE and MCE analyses of peptides and proteins is the prevention of protein adsorption on the inner wall of the capillary.

CE/MCE technology was introduced to improve the efficiency of biomarker protein analysis. This technology can find wide applications in hospitals and other immediate medical facilities. This has been proved by several studies of protein processing related to biomedical research and application. Štěpánová and Kašička [98] and Dawod et al. [6,108] described the developments in protein analysis using various CE and MCE methods in 2011–2017. They showed that sample preparation, preconcentration, inhibition of adsorption and control of EOF, separation by a specific CE/MCE method and improvement of the detection scheme have greatly improved the ability of CE/MCE methods for protein analysis. The innovative application of CE and MCE methods in biopharmaceutical protein quality control, protein determination in complex biometrics, peptide-protein mapping, and determination of physical and chemical parameters of proteins are important achievements in this field.

However, a faster and more sensitive analysis than existing analytical methods is needed.

The application of MF technology in this field is an intensively developed concept to create integrated and fully automated analytical devices that can detect and quantify one or more peptides and proteins from a complex matrix. In this miniaturized MF form of CE, all operations (including sample preparation, derivatization, injection, separation, and detection) are integrated into a micrototal analytical system (uTAS) or lab on chip (LOC) platform [109].

Sonker et al. [110] reported the possibility of using a specific separation system for integrated immunoaffinity extraction to study human serum matrix biomarkers in preterm infants. They used a reactive polymer to immobilize the antibody as a whole and selectively extracted targeted preterm infant markers. For effective separation, they also optimized the MF immunoaffinity extraction protocol and combined it with MCE. The low nanomolar concentration of the two enriched preterm markers in the human serum matrix was studied for 30 min. Their observations may help to develop automated and integrated birth risk assessment tools.

Peptide identification by MS implicitly provides information about post-translational modification (PTM) sites. It can be used to identify the threshold of a particular enzyme under physiologically appropriate conditions. Noach-Hirsh et al. [111] presented a modular integrated MF platform to analyze multiple post-translational modifications of newly synthesized protein arrays. This method can also be used to clarify PTM fingerprints on single cells or tissues. Although the technology is comprehensive, it is limited in size and requires relatively small amounts of biological materials and reagents for research. It is suitable for basic and translational research [111].

More attention has been paid to tools used to identify target proteins or disease biomarkers. The most difficult challenge remains the ability to detect low protein abundances in a single cell. Recent advances in high-resolution/high-quality high-precision mass spectrometers have enabled identifying more than 5000 proteins from less than 100 ng of protein extracts in a short analysis time of 15 min LC–MS [112].

The crude protein content of mammalian cells is only about two orders of magnitude lower than the value that gives a detailed cell extract profile. There is also a need to improve the detection limit of MS, scan rate and intelligent data acquisition technology so that MF proteomics and single-cell or relatively small protein proteomics provide comparable results. Therefore, some researchers believe that MS detection strategies using existing data are most likely to achieve the required performance [112].

There are many important advances in the MF platform that can quantify unicellular proteins. Absolute quantification is the key criterion for the determination of unicellular proteins. Without absolute quantification, it is impossible to accurately compare protein amounts determined by different methods. Research to improve the precision of single-cell protein quantification also has great potential. Fluorescent NPs with higher fluorescence intensity can be used to replace conventional fluorescent probes. Nucleic acid-labeled antibodies can be further amplified and quantified in automated PCR [113].

#### 3.1.2. Evaluation of Polypeptide Antibiotics

Bacterial phospholipid membranes provide a barrier to antibiotics [114]. Host defense peptides, which are part of innate immunity, can destroy these bacterial membranes. Strongly basic or strongly acidic oligo- and polypeptides serve as effective antimicrobial agents. However, quantification of their efficiency is essential for their application in human and veterinary medicine. Traditional techniques for measuring antimicrobial activity rely on microdilution assays to obtain a minimum inhibitory concentration (MIC) value [115,116]. The main disadvantage of this technique is related to the “inoculum effect”, which affects peptide concentrations. Optical methods are the tool of choice in the field of drug discovery and offer a high degree of reliability and efficiency in the determination of antimicrobial activity [117,118].

The MF assay was developed to quantify the “membranolytic activity” of cationic antimicrobial peptides (AMPs) [119]. The “octanol-assisted liposome assembly” (OLA) method [120] was used to obtain ultrahigh capacity MF of giant unilamellar vesicles (GUV) for high-throughput membrane studies [121]. A fluorescence-based readout helped to quantify the activity of AMPs. Cecropin B was the AMP used in the assay, which can induce membrane pores that can enlarge and lead to cell leakage or even disintegration of the bacterial cell membrane. Different concentrations of Cecropin B (0, 2.5 and 5 µM) were used in the experiment with Gram-negative bacteria that ranged in size from 0.15 to 4.2 µm. The results were measured over a period of 10 h. A 50% decrease in signal intensity was representative of vesicle destruction. The researchers measured the drop in intensity to determine whether it was a gradual leakage (pore formation) or an immediate loss of intensity (bursting). Vesicles with a size of 2.5 µm had a mean bursting time of 419.4 ± 3.4 min, and those with a size of 5 µm had a bursting time of 218.3 ± 2.7 min. The activity of the antimicrobial agent was predictable because the bursting time of the vesicles was reduced by half when the concentration of the peptide was doubled. Their method facilitated the study of thousands of vesicles in a single experiment and can be extended to study multiple drug concentrations [116].

#### 3.1.3. Antimicrobial Susceptibility Testing

MF can be used advantageously for antimicrobial susceptibility testing (AST). Microbial resistance to antibiotics is a serious clinical problem, a global public health threat, and the cause of significant complications, adverse treatment outcomes, and mortality [121]. Antimicrobial resistance (AMR) is associated with the overuse and misuse of antibiotics in agriculture and medicine, supported by unregulated over-the-counter sales [122,123]. Modified use of antibiotics, public health measures, and other antimicrobial strategies are desirable to combat the effects of AMR [123,124,125]. The current methods of AST, such as disk diffusion, broth dilution, and epsilometer testing, are inexpensive techniques but require manual liquid handling and pipetting, which consumes time and human resources [126,127]. They also require large sample volumes, cumbersome procedures to interpret results and suffer from false positives. Even recently developed techniques, such as MS, polymerase chain reaction (PCR), genome sequencing or using microspheres, do not address the shortcomings of conventional methods. MF offers promising options for developing point-of-care (POC) systems for the study of AMR, as a single instrument can effectively perform multiple sample processing steps with small samples and reagents. It also provides sensitive, compact, fast and high-resolution analysis with higher sensitivity [128]. For the AMR scenario, MF must provide a self-contained and portable instrument capable of detecting bacteria, performing sensitivity tests, and determining minimum inhibitory concentrations (MIC) within a time frame of a few hours [128]. The key characteristics expected of the device include reproducibility, reliability, ease of manufacture and accuracy. It must also be easy to assemble and available at a lower price than available technologies [129]. Scalability, real-time results, and commercial availability are important aspects of product acceptance. Current systems from MF address the problem of MIK by detecting refractive index changes [130,131,132,133].

MF-based assessment of MIC has been the focus of several research experiments: high-sensitivity optical detection systems with statistical analysis; incorporation of broth-based dilution with MF for rapid testing; and droplets MF, which achieve low contamination, faster mixing, and a high degree of reproducibility [134,135,136,137]. Although AMR-associated MF devices are available, widespread adoption in clinical practice depends on the accessibility of sophisticated devices and the skills required for the protocol [130]. The two main problems in this area are lack of reproducibility and simplicity. Researchers recommend using capillary-driven or paper-based flows MF with a detection system using smartphones, which saves cost and is portable and easy to implement. Signal analysis by smartphones allows timely availability of the result for efficient clinical decision-making [132].

AST has also been the focus of research by Lee and colleagues (2019) [123] through using an integrated MF system (IMS) to assist clinicians in administering appropriate antibiotic therapy to treat bacterial infections. Clinicians typically practice polypharmacy to reduce the likelihood of developing AMR. IMS can assist clinicians in assessing the efficacy of antimicrobial agents by providing important advantages in terms of minimized bacterial and reagent consumption, automation, sensitivity, speed, and increased multiplex capacity. To date, droplet system MF has been used to analyze a given drug and bacteria by isolation [134]. Earlier approaches used an inertial MF chip and RNA detection with hybridization and fluorescence to obtain results [138,139]. They also included “multiple gradient zones” for drugs to assess bacterial growth. Pairs of antibiotics were studied by diffusion [140]. However, these protocols require complex microfabrication, time-consuming operation, dose quantification, and the formation of unstable droplets. The IMS developed in the current study aimed to eliminate these inherent drawbacks of traditional systems. The broth dilution method was used to generate paired concentrations of antibiotics. The goal of the research was to allow clinicians to administer a specific drug early and reduce the overall duration of treatment. The researchers integrated microvalves and micropumps into the MF chip and applied automated liquid handling to combine or dilute antibiotics [126]. Their device was optimized to use less reagent, shorten incubation time, reduce human error, enable rapid liquid manipulation, and achieve significant cost savings. The system was able to assist clinicians in selecting the best option for “empirical treatment” by performing accurate AST for target antibiotics. It proved to be highly reproducible and accurate. The antimicrobial therapy selected using the protocol was able to achieve the optimal bactericidal effect and minimize side effects [125].

#### 3.1.4. Study of Protein–Protein Interactions

Furlan and colleagues [139] presented a workflow that integrates MF with the recognition of MS (MF–MS) to understand protein–protein interactions (PPIs). Hence, far, interactions between different proteins have been studied using various methods, including co-immunoprecipitation, yeast two-hybrid screening, phage display and Western blotting. MF–MS gives good results, and in the absence of antibodies, epitope tagging serves as an alternative method. Proximity labeling is another popular approach [140,141,142,143,144,145,146,147,148]. The method requires only 12,000 input cells, which is a 50–100-fold reduction in the size of current microcentrifuge systems with tube-based workflows. Several optimizations have been achieved by this novel approach: an MF–MS workflow capable of detecting PPIs from small volumes of whole-cell lysate using purification columns in an analogous MF system utilized on-bead protein digestion; their fully automated, small-volume system was successful in reducing variance between experiments; protocol development led to important adaptations, including the exclusion of glycerol during extract preparation and removal of ethidium bromide during extract incubation with beads; their proposed technology may have the potential to detect a smaller number of cells or even a single-cell. The MF–MS system achieved the desired goals of automation and miniaturization. Further improvements in the form of coupling the chip with the systems LC–MS and “direct cell lysis” are desirable to reduce the input effort, optimize time use and achieve positive results in terms of sensitivity and robustness [140].

#### 3.1.5. Proteome Analysis

**Figure 4:**
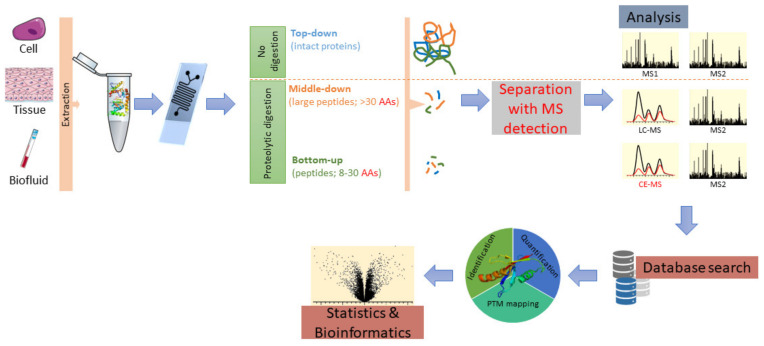
Schematic representation of proteomics approaches.

An important application of MF–MS is proteome profiling. It identifies the complete set of proteins in a sample with the goal of discovering biomarkers for disease diagnosis and therapy monitoring (Figure 4). Proteome profiling is also relevant for determining drug toxicity and is used in the practice of personalized medicine [149]. A major drawback of current proteome profiling techniques occurs during proteolytic digestion of proteins and peptides, which requires long incubation times and high temperatures, leading to oxidation, deamination, and/or nonspecific cleavage [150]. Such homogeneous systems can be replaced by heterogeneous systems to overcome drawbacks, such as autolysis. Moreover, these systems allow using higher ratios of enzymes and substrates, leading to better reaction rates. They are also resilient and stable. Researchers presented a LOC system (heterogeneous digestion system) using DMF in combination with hydrogels for proteome profiling [50,51]. They used gel disks with covalently attached proteolytic enzymes for proteomic sample preparation [150].

Leipert and Tholey (2019) [151] investigated digital MF (DMF) in proteomic sample preparation using bottom-up LC–MS approaches that involve using hydrolysis by proteases to generate peptides. Analysis of sub-nanogram samples is accompanied by challenges at each step of the process: cell lysis, sample purification, protein derivatization and digestion by enzymes, and MS analysis. Sample losses occur due to protein-peptide interactions or low recoveries during desalting operations. Additional risks can occur when handling buffers and solvents containing dissolved reagents and samples and during transfer between reaction vessels. To address these issues, a method for proteomic sample preparation using a DMF chip was demonstrated [50,51]. The process included all major steps, including lysis performed on the chip, extraction of proteins, alkylation or reduction, and digestion by proteases. Optimizations incorporated into their process were protein purification with magnetic beads, use of a single pot for protein removal, improved sample preparation by solid-phase extraction (SPE), and buffer-detergent combinations suitable for microfluidics, digestion by enzymes, and LC–MS analysis [151,152,153,154]).

The choice of polymeric detergent during sample preparation in DMF was particularly important for the success of the experiment [151]. Protein adsorption is a common cause of surface biofouling. Considerable research efforts have been made to investigate using polymeric detergents for the movement of protein droplets in DMF. However, there is limited knowledge of detergents compatible with LC–MS in DMF [155]. In this context, using Pluronic F68 [156] was investigated. This detergent is nontoxic, compatible with cell cultures, aids droplet movement and reduces shear forces. It can be used as an additive for manual loading of samples onto the DMF chip as it prevents cell damage. The detergent Tetronic 90R4 [157] showed the best performance in the process of cell lysis and did not cause droplet pinning. Peptide identification is problematic when using Tetronic or Pluronic detergents due to ionization suppression in MS detection [156,157]. The magnetic bead-based sample cleanup technique (SP3) is compatible with high concentrations of salts, detergents, and chaotropes. SP3 proved to be effective in removing detergents with high molecular mass as there was no “competitive binding of detergents” to the beads during the process of protein aggregation. Moreover, they confirmed that detergents compatible with LC–MS can be useful as antifouling additives for sample preparation methods in proteomics using DMF [151].

Her study was comprehensive and can be used to optimize the sample preparation step for LC–MS analyses. It addresses concerns and introduces optimizations at all stages of sample preparation. The protocol allows droplet manipulation without the aid of robotic pipetting and controls costs. Her primary contributions were in sample cleanup and selection of detergent buffer systems for analysis by LC–MS. SP3 was effective in depleting Pluronic and Tetronic detergents from droplets in samples [156,157]. Another important implication of this research was that detergents compatible with LC–MS were effective in protein digestion in the form of DMF antifouling additives [158]. The researchers used designs with DMF chip electrodes to perform their experiment on nanoliter droplets and have ambitious goals to scale up their research and study protein samples representing single cells. They also plan to use a magnetic lens to allow parallel processing of samples and achieve higher reproducibility in magnetic separation. Another optimization is to record droplet velocity data over longer incubation times to improve detergent buffer function and reduce instrument degradation. They propose to further reduce sample loss by directly connecting LC–MS and DMF or introducing “chip integration of capillaries” [159,160]. In short, their technique has the potential to enable “ultrasensitive proteomics” as a major contribution to the field of bioanalysis [151].

#### 3.1.6. Clinical Applications

Proteomic studies have effectively elucidated the basis of non-communicable diseases for their early diagnosis and monitoring by biomarkers [159]. Several studies have also revealed interesting findings related to potential drug targets [160]. One of the problems often faced in the discovery of plausible biomarker targets is the masking of biomarker targets with low abundance by proteins present in high abundance in biological fluids [161]. This problem can be overcome by using techniques with high sensitivity and specificity, advanced sample preparation, elimination of interference, and high repeatability and reproducibility [157].

MF systems coupled with MS detectors use soft ionization techniques to minimize fragmentation of proteins and other biopolymers. The two main ionization techniques are ESI and MALDI mentioned earlier [162]. The former ESI ionization provides a suitable solution for dealing with complex samples, and the latter offers high sensitivity and tolerance to salts and impurities. MF Protocols used in combination with MS detection can be either droplet MF, digital MF or analog MF [163]. Any of these techniques can be combined to manipulate fluids in microchannels by passive or active pumping. The combination of MF with MS has important applications in translational proteomics, from disease diagnosis and prognosis to disease progression and assessment of therapeutic response [164,165,166]. A number of platforms combining the two approaches have been demonstrated: Capillary sampling, sandwich capillary, and direct substrate analysis for disease diagnosis; parallel emitters, piezoelectric microdispensers, automated droplet microarray spotting, commercial spray tip, tapered corner emitters, and direct substrate analysis for disease progression assessment; and tapered corner emitters, pulled glass emitters, and direct substrate analysis for biotherapeutics monitoring [157,166,167,168,169].

**Figure 5:**
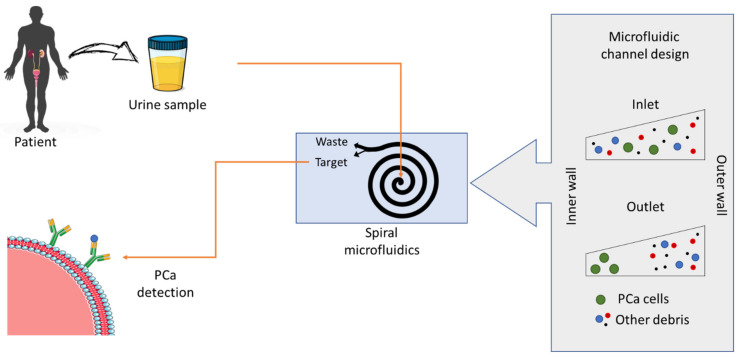
Schematic representation for PCa cell isolation and detection from urine samples resorting to a spiral microfluidic chip. Adapted from https://www.mdpi.com/2072–6694/12/1/81/htm 29 December 2019. Some design variations exist, such as applying inward flow, as opposed to the depicted outward flow. Image partially built using Servier Medical Art images.

Hughes et al., (2012) [170] reported an MF-based protein isoform assay for the purpose of diagnosis. They used a multistep single-channel immunoblotting strategy to separate and immobilize proteins and apply antibody probes. They used a “quantitative protein isoform assay” by integrating MF in combination with a photoactivatable 3D gel and electrophoretic control and achieved a pump-free process. Results showed a five- to fifteen-fold improvement in the time required to perform the assay, with isoform levels available in 80–120 min after analysis. Their method outperformed capillary immunoblotting and western blotting. The improved performance was achieved by better mobilization of proteins due to the higher availability of reactive sites and by using nanoporous gel for electrokinetic transport. As a result, the capture efficiencies achieved with the protocol were two to three times higher compared to existing approaches. The method was simpler than the ELISA approach, which uses matched pairs for antibody capture and detection. In contrast to the ELISA approach, the protein isoform assay used here used only a single primary and secondary antibody for analysis [170]. Chen et al. [171] applied a different MF approach to circulating endothelial cells (CECs), which are widely reported as promising biomarkers of endothelial damage/dysfunction in coronary artery disease (CAD). It involves using spatially staggered micropillaries to distribute the trapped cells to all parts of the collection chamber and prevents the accumulation of flow debris. This results in a clean and unobstructed field of captured cells, facilitating real-time monitoring of flow conditions and enabling on-chip immunofluorescence staining processes for more accurate identification results. In addition, the unobstructed capture chamber enables continuous maintenance of flow rates during the label-free assay process, which is in stark contrast to the frequent flow blockages in the highly congested debris field of the membrane system. Jones et al. [172] developed a microfluidic immunoarray to measure an 8-protein panel in serum that can identify patients with prostate cancer and inform the need for biopsy. This 8-biomarker panel contains both common prostate cancer biomarkers and proteins specific to aggressive and metastatic forms (Figure 5). Proteins at clinically relevant concentrations with good accuracy and sensitivity and low fg/mL limits of detection (LODs). High sensitivity was traded for speed, allowing analysis of a 100-fold diluted serum sample in 40 min. Data from 130 serum samples from prostate cancer patients identified single proteins with good specificity and sensitivity of 84% for differentiating between moderate and ≥8 biopsy Gleason scores.

Although the MF–MS combination offers a novel alternative to traditional peptidomics and proteomics analyses, its clinical application is still limited. This is because MF devices still need complex manufacturing processes, require considerable resources and equipment, and are complicated in operation [172,173]. It also requires extensive statistical analysis and cost, making it difficult to integrate seamlessly into clinical practice [131]. Another approach, the working principle of the device, was a sandwich immunoassay with in situ Raman detection. The Raman microchip included four inlets: a mixing chamber, a valve, a Raman detection chamber, and an outlet. The exosome sample and anti-CD63 magnetic beads were mixed and reacted as they flowed together through the staggered triangular micropillary mixing chamber. The Mag-CD63 exo-complex was magnetically fixed on the Raman detection region. The entire analysis was performed using only 20 μL of the exosome sample in 1 h [174]. Peddle and colleagues (2017) described the culmination of MS-based approaches and MF for disease monitoring and therapeutics and identified regulatory and client approval as key factors for technology adoption into clinical practice. In addition, customer and market adoption requires significant operational performance gains and economic feasibility. The devices must also be able to be integrated into existing workflows and hardware systems. Finally, developing these novel technologies requires investor confidence and extensive staff training [157].

### 3.2. Separation and Analysis of Cells

#### 3.2.1. Cell Sorting and Single-Cell Analysis

Sorting cells based on their size, type, or density is important for their study and analysis, especially for distinguishing diseased from normal cells in the diagnosis and treatment of various diseases [17]. Cell separation methods are evaluated based on their separation efficiency, enrichment, and throughput [175]. Separation efficiency refers to the homogeneity of the separated cell (sub)populations, enrichment refers to the increase in cell concentrations compared to the initial concentration, and sample throughput refers to the speed of cell separation. Fluorescent markers and labeling techniques have been an important aspect of conventional approaches. This problem was addressed by relying on the physical properties of the particles that acted as targets for separation.

One of the most important features of MF devices is their ability to separate and sort cells for automated processing in the diagnosis and treatment of disease [176,177]. Passive MF devices are preferable to active devices for several reasons. Active devices use variable forces, including magnetic, electrical, and optical forces, for cell movement and operate with higher efficiency [176]. However, they are more expensive and can damage the cells. On the other hand, passive systems work with inertial forces, sediment, gravity, and filters for cell enrichment. The design factors of passive devices include the elements of viscosity, capillary forces, and surface forces. Since they do not require extensive equipment, improvements in their efficiency can lead to higher reliability [178,179]. In this context, flow cytometry is an important strategy for sorting cells based on their characteristics, shape and size in heterogeneous cell populations. Separation by membrane filtration and centrifugation has inherent problems related to preparation complexity, time and skill requirements. In contrast, methods from MF offer advantages in terms of high speed of analysis and low cost. They are also less invasive. Among the three different groups of separation techniques (active, passive and combined), the passive method is the simplest. Cell separation in MF requires some kind of fractionation method or force [180]. The different types of filters used for this purpose include microscale filters, which sort cells based on their deformability and size, and hydrodynamic filtration techniques, which take into account the size and shape of the cells. Other separation techniques include pinched-flow fractionation, which separates particles based on their size, and the deterministic lateral shift method, which changes the trajectories of cells based on their size [181]. Gravity and sedimentation fractionation methods are based on the density of particles. Biomimetic devices separate particles based on their actual behavior in the human system. Finally, inertial devices can be used for particle migration [178,181,182].

For cell separation systems, the separation capacity takes into account the amount of sample available for analysis, the distinguishing characteristics of the cell types, the purity required, and the desired characteristics of the isolated population [183]. The total number of cells lost during the separation process, the viability of the cells after separation, and the physical pressure on the cells. Finally, the choice of effective sample preparation procedures, the time required for the entire cell separation process, and the cost-effectiveness of the technology are also important [54]. It is advantageous to integrate the technology for the separation of cells and other particles into the lab-on-chip equipment because these unlabeled processes are continuous, the separation process can be continuously monitored, and the sample components can be moved laterally so that each part can be collected independently [184]. It is expected that the specific cells in the biopsy sample will be treated individually. Further analysis should focus on system expansion, which obviously requires thorough optimization [110].

MF design considerations were related to the design and generation of forces and determining how the forces move the particles. A discrete-phase approach was used to determine particle motion and to study the effects of forces, such as buoyancy force, virtual mass force, pressure gradient force, Saffman force, Basset force, body force, and Brownian force [185]. High throughput methods MF included “controlled incremental filtration”, “continuous particle separation in a spiral microchannel,” and “shear modulated inertial migration”. Data analysis was performed using MATLAB software [186,187]. In the study, three different passive MF methods were demonstrated for stepwise filtering of fluid from the main channel into the side channels to achieve single-cell isolation. The method that proved most successful had low recovery [178].

Ahmad et al. proposed a tapering MF device for separating multiple particles (cells and microbeads) in biological samples (removing contaminants from heterogeneous mixtures) in a passive process [188,189]. Their microfabrication research focused on heterogeneous samples and addressed the problems associated with the isolation of cells and microbeads. The present research involved developing a microfluidic device capable of using passive separation technology for microparticles and cells. The device consists of a tapered microchannel with an outlet, an inlet, and tapered angles. Sample solutions are introduced into the inlet and move along the centerline of the narrow microchannel, which serves as the focal region. The cells and microbeads move along different trajectories as they leave the focal area. These trajectories are affected by the density, deformability, and size of the cells. Particles and cells migrate laterally depending on their flow velocity and radius. The widening of the microchannel causes the average velocity of the particles and cells in the center to slow down. This change causes the flow to form a downward angle near the lateral exits. This creates streamlines that help the particles and cells travel to the collection outlets. Inside the microchannel, the particles and cells experience hydrodynamic drag that is introduced into the fluid flow. Particles and cells entering the microchannel cause a change in flow velocity at the outlet. The widening of the microchannel results in varying degrees of hydrodynamic drag at different outlets. The hydrodynamic drag at an outlet causes trapping. The design of the microchannel is optimized using simulation finite element analysis (FEA). The system MF created with hydrodynamic principles is valid for many different applications. Its parameters and operating conditions can be further optimized. Calibration of parameters, such as flow rate and sample concentration, helps to increase the throughput significantly [189].

The major challenges for MF separation devices are the throughput of the separation process, complexity in design and purity of the sample. The solution proposed in the paper achieves the desired results by using multiple cone angles to separate samples with high purity. The “hydrodynamic separations” are supported by a coupling mechanism and sedimentation, which help to achieve the required sample purity. The process is supported by technology in the form of computer-aided design (CAD) [190,191] and finite element analysis (FEA) [192,193]. The device is fabricated using soft lithography.

#### 3.2.2. Secretome Analysis and Single-Cell Omics Analysis

**Figure 6:**
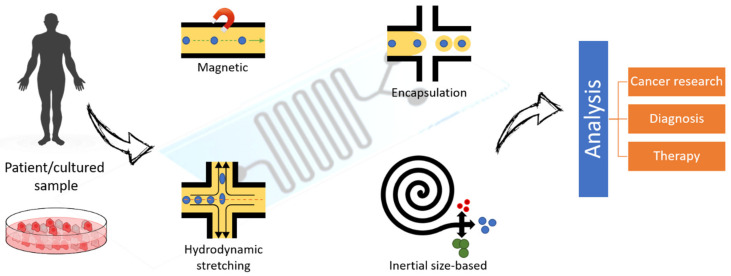
Schematic representation of the most commonly used techniques for the isolation, trapping and manipulation of single cells in a microfluidic device. Potential scope of applications is highlighted.

**Figure 7:**
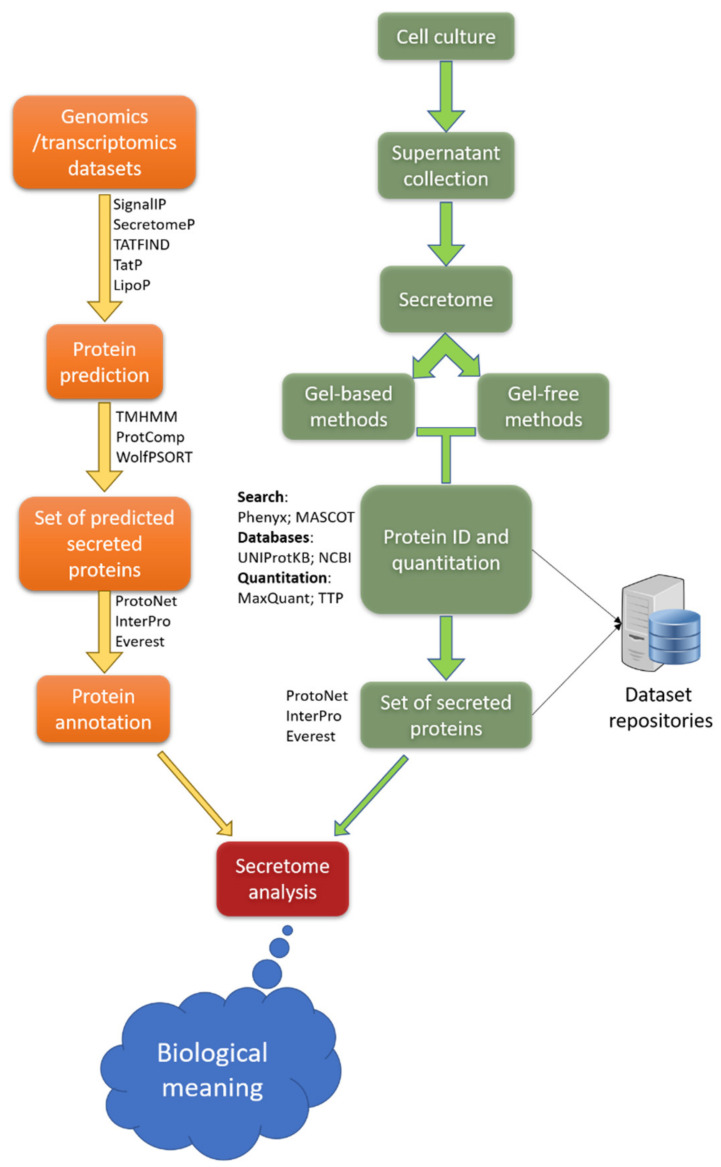
Schematic representation of the workflow of secretome analysis for the detailed characterization proteins secreted by different cells, tissues and organs. The two main approaches described, as well as the most commonly used bioinformatics tools used in each of the shown steps. Adapted from https://www.sciencedirect.com/science/article/pii/S1570963913000502, 26 November 2013.

The cell secretome is a complex proteome secreted by cells. It is the basic mechanism for communication between cells in vivo and in vitro. Analysis of proteins secreted in body fluids can determine biomarkers for important pathophysiological conditions [194]. However, due to the complexity of protein content in body fluids, there is a great need for a better understanding of the proteins secreted by different cell types. This can be more easily explored in vitro (Figure 6). To this end, MF tissue culture systems may be particularly important as they can accumulate endogenous and exogenous signals at the microliter scale, thus better preserving the self-regulation that occurs in small interstitial spaces in the body [195] (Figure 7).

Hu et al. [194] quantitatively analyzed the proteins secreted by human foreskin fibroblasts grown in multi-well plates or in MF systems. This comparison showed the general accumulation of secreted proteins in MF systems. However, not all proteins accumulate equally. This suggests that the culture microenvironment plays a feedback role in cell regulation of protein secretion. Therefore, it is important to study the cell secretion group in a culture system that is more similar to the conditions of the microenvironment in vivo, and the MF volume is small, which better mimics the small interstitial spaces in the tissue [195].

Heterogeneity studies based on single-cell-omics analyses are important for identifying diverse cell populations, discovering new cell types, revealing informative cell properties, and discovering important relationships between cells. Recently, MF has become a powerful technique for single-cell-omics analyses due to the MF technology’s advantages of flow, sensitivity, and precision [196]. Here, recent advances in MF single-cell oocyte analysis, including various designs of microfluidic platforms, lysis strategies, and oocyte analysis techniques, have become essential biomolecular tools [196,197].

Compared to traditional test tube operations, MF offers excellent flow, sensitivity, precision, integration, and partial automation in omics research. However, the new research still faces challenges [198]. First, in single-cell isolation, not all advantages, such as high throughput and effective cell isolation, simple chip design and fabrication, and integrated multistep operation, can be maintained because each isolation method has its advantages and limitations [196,197]. Second, in omics analysis, barcoding technologies used for cell/molecule labeling can increase throughput and significantly reduce cost, but they are often biased (e.g., the 3-terminal cDNA ends) and can lead to loss of important signals. To address this challenge, mature third-generation sequencing technology can reverse this trend by increasing read length and single-molecule sequencing [196,197].

#### 3.2.3. Investigation of Stimulus-Driven Cell Behavior

Lazar and colleagues [199] attempted to elucidate cell behavior by using MF in combination with MS analysis. Their research aimed to perform proteomic profiling for cell responses to stimuli. Their motivation was the complex nature of cell responses to chemical or physical stimuli: different time scales and rates, presence or absence of protein synthesis; transient or sustained signaling processes; involvement of neuronal, metabolic, and immune systems; involvement of signal transduction and protein phosphorylation; and the effects of cell proliferation, growth, transformation to a disease state, and differentiation [200,201]. Their approach consisted of important features, such as including a substantial number of cells for plausible MS analysis and achieving rapid sampling of cell contents and uniform cell stimulation. Their protocol was designed to deliver the cell stimulant axially and transversely. Fluorescent dyes were used to identify fluid manipulation on the chips and determine the results of stimulation. Different biological processes were addressed by axial and transverse delivery. Processes that required sustained stimulation of cells were not affected by a small concentration gradient. Those that did not require a concentration gradient were amenable to axial delivery. In contrast, biological processes characterized by rapid responses to stimulation, those requiring steady and rapid delivery of the stimulant, or those requiring rapid lysis of cells were amenable to transverse delivery of the stimulant [199,202].

#### 3.2.4. Investigation of Biomolecular Coronas Nanoparticles

Nanoparticles (NPs) serve as potential vehicles for drug and nucleic acid research. However, their mechanism of action has not been fully elucidated. The implications of this limited understanding of in vitro data mean that the physiological response to NPs in vivo cannot be predicted. Biomolecular coronas (BCs) formed on NPs by their contact with biofluids are of great importance in molecular medicine [203,204]. BCs are influenced by various factors, such as size, temperature, surface charge, hydrophobicity, incubation time and protein source. This premise is further explored to associate disease-specific protein changes with BC composition to gain deeper insight into personalized BCs. BCs may be able to distinguish cancer patients from normal patients and understand disease progression. The current research is focus on discovering new mechanisms to generate crowns through applying shear stress [203,204]. Previously, the process of incubating NPs with human serum (HS) or human plasma (HP) was used to adsorb blood proteins. Recent research shows that the sheer stress of a “laminar fluid flow” compared to static incubation leads to the formation of more complex coronae [205,206,207]. It is expected that this form of dynamic flow will provide a better understanding of actual physiological processes. MF provides a standardized environment for performing dynamic incubation protocols as the reproducibility is far greater than conventional silicone tubing and peristaltic pumps [204].

Digiacomo et al. [204] investigated the role of MF in this field by using gold nanoparticles (GNPs) to explore their plausibility in chemotherapy research and drug delivery in cancer. GNPs can activate multiple juxtaposed receptor sites and increase cellular uptake. They possess surface plasmon resonance and can efficiently convert light to heat, localize the effect of temperature, and induce rapid cell death. The analysis showed that the sheer stress of an MF environment leads to the formation of more negatively charged NPs and affects protein composition. Future meta-analysis will be possible by standardizing the composition and formation of BCs [203,204]. Changing the size and shape of GNPs may lead to different results [208]. It is important to note that the method chosen to analyze the particles MS/MS is not able to identify the exact protein structure on the surface of GNPs. It may be possible to understand the role of NPs in the physiological response by studying the exposed protein epitopes. In addition, a detailed understanding of the structure of the corona is essential to determine the nature of activation of processes at the cellular and subcellular levels [204].

## 4. The Future of Microfluidics

**Figure 8:**
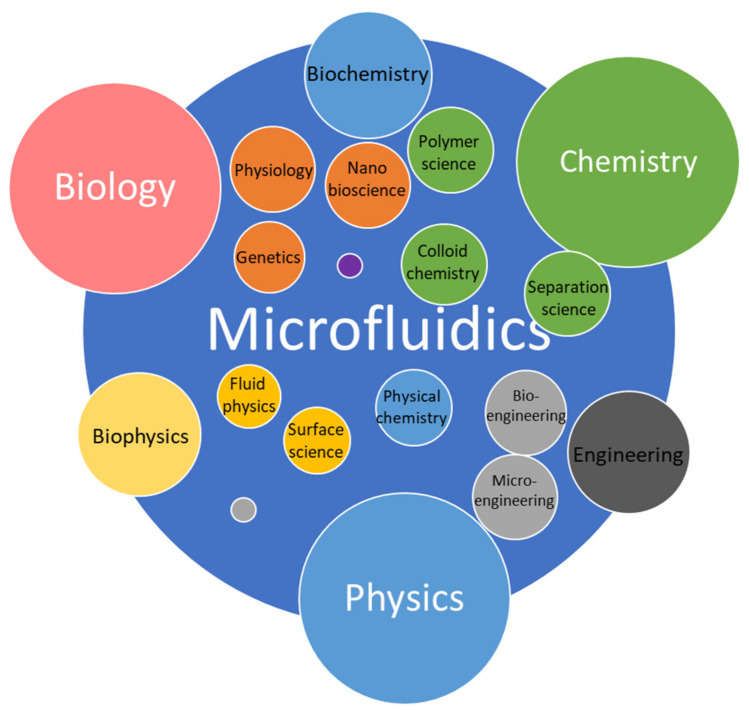
Microfluidics shows a wide range of applications, allowing fundamental studies of processes and systems at the molecular-scale in science and engineering, exhibiting the potential for developing new systems and technologies for an ever-growing list of applications at the interface of different fields of research, namely, physics, chemistry, and biology.

Further intensive developments and applications of MF technology are expected in many fields in the near future (Figure 8). MF systems are considered high-throughput devices but are not yet sufficiently fast or suitable for continuous, repeated use. Nevertheless, MF is revolutionizing the way “liquids” are handled in almost all fields and applications. Defense, special operations/intelligence, renewable energy, medicine, physics, chemistry, biochemistry, biology, biotechnology, green technology, and electronics are some of these applications. Several groups have demonstrated the integration and miniaturization of various core genetic analysis functions into the autonomous integrated fluidic chips. This will promote the spread of healthcare to the public. It will enable independent, autonomous and decentralized control of public health and monitoring of biological threats [209].

This can be realized in many ways, such as health kits that provide therapeutic and preventive medications to the elderly; regular health checks will be possible with minimal samples; patients with chronic diseases that need monitoring will have home testing systems; lab-on-a-chip (LOC) systems will allow tracking and alerting of at-risk patients. Microchips could also be deployed in public places and used to detect outbreaks of new infectious diseases or early signs of biochemical terrorism [210]. MF could play a significant role in all of these scenarios in the future, as well as others not currently imagined.

Other examples of such advances include artificial cells, synthetic “hybrid” biomolecules, and synthetic biomaterials. Future products that embody “inner space” will include artificial organs and nanoparticles that can detect and target disease [211]. Potential applications could include on-chip cell and tissue monitoring, drug discovery, and rapid clinical diagnostics. One area of particular interest is the design of new protein catalysts for industrial applications. For in vivo neurochemistry applications, improvements could be made to the nESI–MS method to provide greater coverage and sensitivity. The nanoESI–MS assay could also be paired with in vivo microdialysis to enable measurements with a high time lag using a conventional sampling method. The success of the optimized techniques will ultimately depend on consumer acceptance of the MF technology and regulatory approval for widespread use in clinical practice. Several other factors play a role in integrating the technology into mainstream bioanalytics, including investor confidence and staff training, as well as the speed and development of low-cost MF manufacturing facilities in the future [210,212]. Moreover, advanced computational methods are needed to extract knowledge from the generated datasets. Analysis of biological data has historically been the focus of the field of bioinformatics. This is due to the combined use of expertise in biology, computer science, statistics, mathematics, and engineering, and other fields to analyze and interpret biological data, often in developing software and methods to process, store, and analyze large amounts of data. Although this is traditionally done using mathematical and statistical methods, analysis is typically based on methods from statistics and artificial intelligence (AI). In recent years, advances in AI have meant that the field of bioinformatics has seen dramatic progress thanks to new developments in AI, with profound implications, particularly for the field of pathology. Indeed, AI makes use of statistical, computational and mathematical capabilities. AI requires appropriate training datasets and algorithms to improve results before testing, similar to traditional statistical methods [213,214,215,216,217]. AI focuses on building automated decision systems, unlike traditional statistical approaches that rely on rule-based systems [215,218]. AI methods are categorized into three types of learning techniques: supervised, unsupervised, or semi-supervised or augmented learning. It is beyond the scope of this article to explore these three forms of learning approaches in detail. Instead, we focus on supervised learning as this is the most commonly used method in biopeptide prediction [217,219]. Supervised learning methods are used to build and train prediction models for data category values or continuous variables. Unsupervised approaches are used to build models that allow the clustering of data without any user requirements. Recent developments in high-performance sequencing technology indicate that unprecedented amounts of protein sequencing data will be generated in the coming years. This underscores the need for in silico programming to enable rapid and reliable measurement of novel biopeptides by many applicants. Sequence-based AI approaches can be used for selection and testing in a wet-lab experiment to refine the design of therapeutic peptides before they are synthesized. This complicates their usefulness to the end-user and highlights the need to systematically evaluate and develop these approaches, taking into account the state of the art in methodology and predictive performance [216,218].

## 5. Conclusions

MF is advanced high-throughput microtechnology for the separation and analysis of peptides, proteins and other biomolecules and cells with the potential to contribute significantly to the generation of new relevant knowledge in the fields of physics, chemistry, biochemistry, molecular biology, biotechnology and medicine. MF is the platform of choice for researchers as it offers important advantages in terms of miniaturization, automation and integration. In addition, reagents and samples are required in extremely small quantities and volumes, leading to developing microchips for applications targeting cell responses to stress, immunoassays and interactions between cells, cellular signal transduction and tissue-based studies, with corresponding resource and environmental benefits. MF provides the complexity required for analysis of both the proteome and peptidome. The small molecular mass of peptides and their availability in small quantities in samples make it particularly relevant for peptidome studies. MF also has demonstrated its potential for better interpretation of biological results by enabling an environment for cell populations that integrates important aspects of conventional biological processes. Miniaturization is a key advantage that allows MF to be applied to extremely small samples and even single cells. However, despite its potential, MF technology is still in its infancy, and extensive research is required to fulfill the promise of this applied approach.
